# Cytokines and adolescent anorexia nervosa: a case-control study

**DOI:** 10.1186/s12888-026-07874-9

**Published:** 2026-02-03

**Authors:** Nikola Ferencova, Zuzana Visnovcova, Igor Ondrejka, Igor Hrtanek, Dana Funakova, Ingrid Tonhajzerova

**Affiliations:** 1 Department of Physiology, Jessenius Faculty of Medicine in Martin, Comenius University in Bratislava, Mala Hora 4C, Martin, Slovak Republic; 2 Biomedical Centre Martin, Jessenius Faculty of Medicine in Martin, Comenius University in Bratislava, Mala Hora 4D, Martin, Slovak Republic; 3 Psychiatric Clinic, Jessenius Faculty of Medicine in Martin, Comenius University in Bratislava, University Hospital Martin, Kollarova 2, Martin, Slovak Republic

**Keywords:** Anorexia nervosa, Cytokines, Pro-inflammatory/anti-inflammatory balance, Adolescent age, Depressive symptomatology

## Abstract

**Background:**

Anorexia nervosa (AN) represents a relatively common eating disorder associated with the highest mortality from all mental disorders. However, the underlying AN psychobiology remains unclear. The disruption in immune pathways seems to play an important pathophysiological role in AN. Therefore, this study aimed to evaluate the cytokine profile in association with assessing the pro-inflammatory/anti-inflammatory balance in drug-naïve adolescent AN girls in the early stage of the disease.

**Methods:**

Selected cytokines including interleukin (IL)-1α, IL-1β, IL-2, IL-4, IL-6, IL-8, IL-10, interferon-gamma, tumor necrosis factor-alpha (TNF-α) were evaluated in 35 adolescent AN girls (mean age: 15.0 ± 1.6 years) and 25 age-matched control adolescent girls (mean age: 15.9 ± 1.5 years). Moreover, selected cytokine profiles and cytokine ratios were calculated to evaluate the pro-inflammatory/anti-inflammatory balance in adolescent AN. The presence and severity of depressive symptomatology were evaluated by full-filling the standardized and validated Children´s Depression Inventory.

**Results:**

The plasma levels of IL-1β, IL-6, TNF-α, M1 profile, pro-inflammatory profile, Th1/Th2 ratio, Th1/Treg ratio, Th1/Th2 + Treg ratio, and pro-inflammatory/anti-inflammatory ratio were significantly higher in AN adolescent girls compared to controls (*p* = 0.002, *p* = 0.009, *p* = 0.031, *p* < 0.001, *p* = 0.042, *p *= 0.031, *p *= 0.021, *p* = 0.016, *p* = 0.001; respectively). On the other hand, the plasma level of IL-1α was significantly lower in AN adolescent girls compared to controls (*p* = 0.005). Moreover, BMI predicted levels of IL-4 and IL-1β (*p* = 0.009, *p* = 0.046, respectively), and depressive symptomatology predicted level of IL-8 (*p* = 0.012) in adolescent AN girls. Lastly, ROC analysis revealed a certain predictive value of significantly changed cytokines, cytokine profiles, and ratios in adolescent AN, which markedly improved by the combination of these parameters (AUC = 0.930). Moreover, cross-validation analysis of the combined model AUC using the K-Nearest Neighbors model achieved a high overall accuracy of 0.917 for both groups, with a precision of 1.000 for the AN and 0.800 for the controls.

**Conclusions:**

Adolescent drug-naïve AN in early stages of the disease is characterized by enhanced IL-1β, IL-6, and TNF-α signalling indicating the activation of pro-inflammatory response system. Whether these inflammatory abnormalities are AN-specific or related to other confounding aspects of the disease still needs to be resolved, since BMI and depressive symptomatology contributed to the alterations in evaluated inflammatory markers in this study.

**Clinical trial number:**

Not applicable.

## Background

Anorexia nervosa (AN) represents a serious mental disorder characterized by abnormal eating behavior, low body weight, distortion of body image, and fear of weight gain [1]. AN significantly impairs physical and psychosocial functioning and is associated with an extremely high mortality rate [2]. The treatment of eating disorders including AN is less effective when compared to other mental disorders, particularly due to still widely unknown pathological mechanisms underlying the disorder. In this context, the emerging research suggests the potential role of immune system disturbances in the AN pathophysiology [3]. More specifically, the physiological balance of pro-inflammatory and anti-inflammatory pathways seems to be altered in AN confirmed by several cross-sectional studies as well as meta-analyses revealing different cytokine profiles in AN patients compared to healthy controls [3–7]. Cytokines are important immunomodulators and neuromodulators regulating the neuroimmune responses *via* complex networks and are characterized by pro-inflammatory and/or anti-inflammatory properties. The most important cytokines associated with predominant pro-inflammatory activity include interleukin (IL)-1β, IL-6, tumor necrosis factor alpha (TNF-α), and interferon gamma (IFN-γ) [8]. From AN perspective, the pro-inflammatory cytokines can act on the central nervous system (CNS) leading to changes in cognition, mood, and behavior [9] including inflammation-related anorexia and reduced intake of food [10]. In this context, the most recent systematic review reported IL-6 and TNF-α as the most promising signaling pathways potentially involved in AN pathogenesis [3]. On the other hand, only a few studies simultaneously evaluated the levels of anti-inflammatory cytokines potentially counter-regulating the heightened pro-inflammatory activity reporting decreased levels of transforming growth factor-beta [7, 11] and decreased or unchanged levels of IL-10 [7, 11–13] in AN patients compared to healthy controls. Importantly, the majority of so far studies have reported a low-grade pro-inflammatory state in AN patients at adult age. However, AN usually manifests already during adolescence [14]. Since AN adolescents experience shorter durations of the disorder and require fewer treatment interventions, the evaluation and better understanding of the potentially altered cytokine profiles at this developmentally sensitive life period is crucial to reveal the fundamental mechanisms involved in the AN pathophysiology. Therefore, the primary aim of the study was to evaluate pro-inflammatory and anti-inflammatory cytokines in association with the assessment of the pro-inflammatory/anti-inflammatory balance in drug-naïve adolescent AN girls in the early stage of the disease.

Further, it is well-known that the body composition of AN patients is changed compared to that of healthy individuals [15, 16]. In this context, the cytokines´ levels can be influenced by several factors including anthropometric variables [7, 17, 18]. However, from this perspective, the most of mental disorders are associated with increased body mass index (BMI) compared to healthy individuals [19], whereas abnormally high BMI has been suggested to be strongly correlated with pro-inflammatory response confounding the relation between mental well-being and inflammation [20, 21]. Contrary, AN as a specific mental disorder represents a unique opportunity to assess the impact of abnormally low BMI on inflammatory pathways which has been rarely studied so far. Therefore, another aim of the study was to evaluate the impact of BMI on cytokine levels, profiles, and ratios in adolescent AN girls.

In addition, there is a clinically relevant comorbidity between AN and major depressive disorder (MDD) already in adolescence [22]. Since adolescent MDD is reported to be associated with abnormal cytokines´ profile compared to healthy controls [23–25], it remains questionable whether the cytokine changes hypothesized in AN adolescent patients can be caused by the disease itself or comorbid disease´s symptoms. Thus, the final aim of the study was to assess the potential impact of depressive symptomatology evaluated by self-administered standardized questionnaire on cytokine levels in adolescent girls with AN.

## Methods

This study was approved by the Ethics Committee of the Jessenius Faculty of Medicine in Martin, Comenius University in Bratislava (EK69/2018) in accordance with the Declaration of Helsinki (2000) of the World Medical Association. All participants and their guardians were carefully informed about the study protocol and gave informed written consent before participation in the study.

### Subjects

**Fig. 1:**
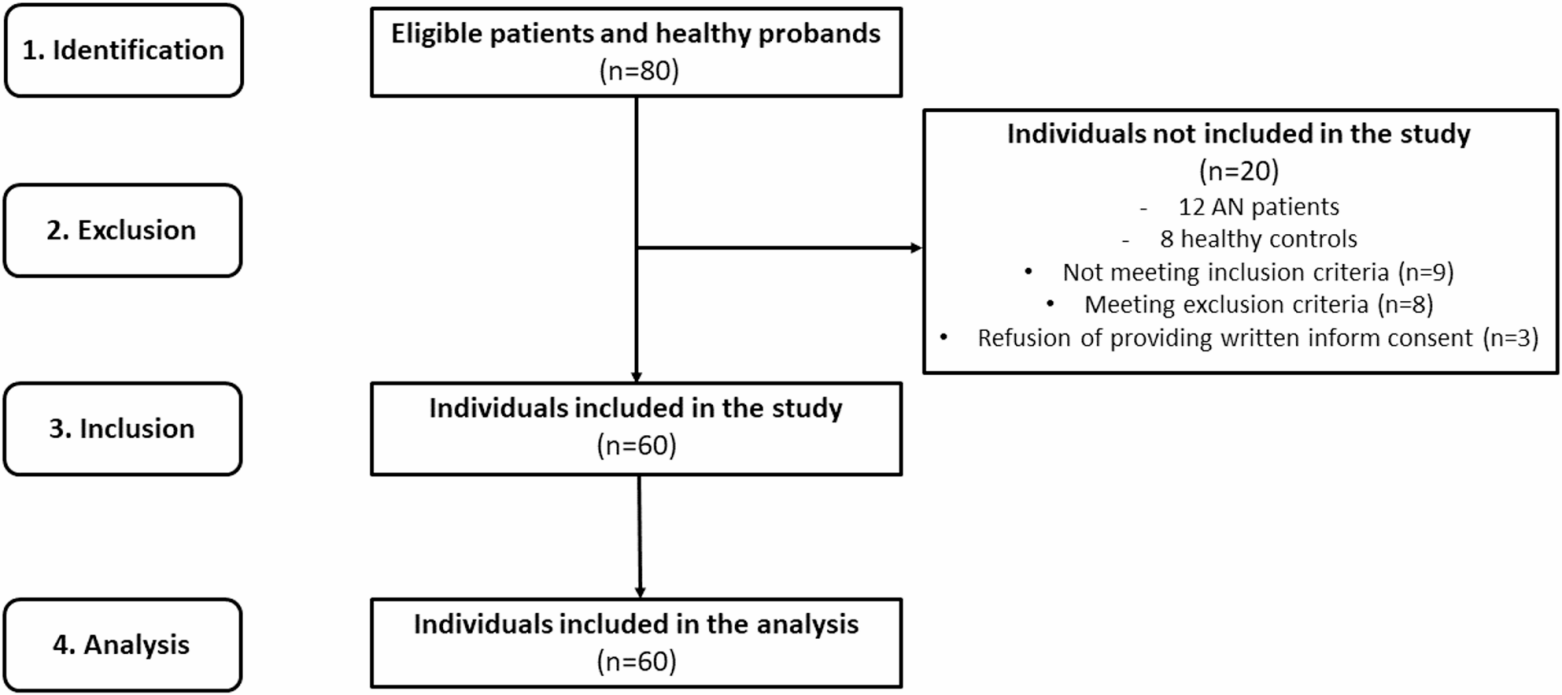
Study flow chart. AN – anorexia nervosa

The final study cohort consisted of 35 adolescent girls suffering from AN (mean age: 15.0 ± 1.6 years, BMI: 16.2 ± 2.5 kg/m^2^), recruited from the inpatients admitted to the Psychiatric Clinic of Jessenius Faculty of Medicine and University Hospital in Martin and 25 control adolescent girls matched for age (mean age: 15.9 ± 1.5 years, BMI: 20.6 ± 2.4 kg/m^2^) (Fig. 1). We have included only female patients diagnosed with AN, aged between 12 and 18 years. The diagnosis of AN was assessed by a thorough clinical investigation based on an unstructured diagnostic interview by a child/adolescent psychiatric specialist according to the Diagnostic and Statistical Manual of Mental Disorders, DSM-5 [26]. The AN patients were examined during the first days of hospitalization before starting any pharmacological and non-pharmacological interventions. Control girls were normal weight and had never been treated for any mental disorder. Exclusion criteria applied for both groups were following: acute and chronic infections, allergies, endocrine diseases, immune diseases, and systemic diseases determined by self-report or doctor’s report as well as history of psychiatric, immunomodulatory, and analgesic/anti-inflammatory/antibiotic treatment.

### Blood collection and cytokine determination

Fasting peripheral venous blood samples (5 mL) were collected into EDTA test tubes in the morning. Next, the blood samples were centrifuged for 15 min at 2500 rpm and 4 °C (refrigerated centrifugation, Hettich Universal 320R, Tuttlingen, Germany), and the obtained plasma was kept frozen at -80 °C until the analysis. Selected cytokines (IL-1α, IL-1β, IL-2, IL-4, IL-6, IL-8, IL-10, IFN-γ, TNF-α) were analyzed and quantified using a biochip array technology (Evidence Investigator, Randox, United Kingdom) following the manufacturer´s instructions. Next, raw cytokine data were logarithmically transformed and consequently used for the calculation of z-unit weighted composite scores [27, 28] according to the formula: z = (x – M)/SD where z represents standardized score, x represents raw cytokine level of the proband, M represents mean level of the cytokine in the combined (i.e. AN + control) group, and SD represents standard deviation of the level of cytokine in the combined group. Obtained data were used to determine individual cytokine profiles such as Th1 profile (i.e. zIL-2 + zIFN-γ), M1 profile (i.e. zIL-1β + zIL-6 + zTNF-α), pro-inflammatory profile (i.e. zIL-1α + zIL-1β + zIL-2 + zIL-6 + zIL-8 + zIFN-γ + zTNF-α), and anti-inflammatory profile (i.e. zIL-4 + zIL-10), and selected cytokine ratios such as Th1/Th2 ratio (i.e. (zIFN-γ + zIL-2) – zIL-4), Th1/Treg ratio (i.e. (zIFN-γ + zIL-2) – zIL-10), Th1/Th2 + Treg ratio (i.e. = (zIFN-γ + zIL-2) – (zIL-4 + zIL-10)), and pro-inflammatory/anti-inflammatory ratio (i.e. (zIL-1α + zIL-1β + zIL-2 + zIL-6 + zIL-8 + zIFN-γ + zTNF-α) – (zIL-4 + zIL-10)). The subtraction in ratios was performed due to logarithmically transformed data. We have used similar analysis of the selected cytokines, cytokine profiles, and cytokine ratios in our previous studies (for details see [23, 29]).

### Children´s Depression Inventory (CDI)

The presence and severity of depressive symptomatology were evaluated by completing the CDI according to the author´s instructions. The inventory measures different components of depression including anhedonia, ineffectiveness, interpersonal problems, negative mood, and negative self-esteem. The CDI contains 27 items with each item having three statements and the child/adolescent selects the one from the statements which best describes their feelings during the past two weeks. Each item is scored on 0 (absence of symptom) to 2 (definite symptom) scale with the range of possible total score 0–54 and a higher CDI total score represents a higher depressive state [30].

### Statistical analyses

The data were explored and analyzed in jamovi version 1.2.27 (Sydney, Australia). The Shapiro-Wilk normality test was used for the evaluation of data distributions (Gaussian/non-Gaussian). The between-group comparison of the Gaussian-distributed variables was performed by the Student´s t-test and the between-group comparison of non-Gaussian-distributed variables was performed by the Mann-Whitney test. The associations between BMI, depressive symptomatology and evaluated parameters in adolescent AN girls were analyzed using Spearman ´ s rank-order correlation test with Bonferroni correction to minimize the family error rate in multiple comparisons. Besides, the generalized linear model analyses were used to evaluate the impact of BMI and depressive symptomatology on the cytokines´ levels in AN adolescent girls. In addition, a ROC curve analysis was performed to determine the AUCs, sensitivities, and specificities of the identified parameters to distinguish AN and controls. The DeLong test was used to determine if the difference between the AUC values of predictive models is statistically significant. Lastly, a K-Nearest Neighbors (KNN) classification algorithm was implemented in JAPS 0.95.3 (Netherlands) to distinguish between individuals in the AN and control groups, with the model optimized for maximum validation accuracy. Power analysis (effect size) was performed post-hoc in G*Power 3.1.9.4 (Dusseldorf, Germany) using parameters: difference between two independent means, two tails, α = 0.05, and sample size due to the real group size. Data are expressed as mean ± SD or median (interquartile ranges). A value of *p* ≤ 0.05 (two-tailed) was considered statistically significant.

## Results

### Evaluation of the cytokines, cytokine profiles, and cytokine ratios

The plasma levels of IL-1β, IL-6, and TNF-α were significantly higher in AN adolescent girls compared to control adolescent girls (*p* = 0.002, *p* = 0.009, *p* = 0.031; respectively). On the other hand, the plasma level of IL-1α was significantly lower in AN adolescent girls compared to controls (*p* = 0.005). Moreover, the M1 profile, pro-inflammatory profile, Th1/Th2 ratio, Th1/Treg ratio, Th1/Th2 + Treg ratio, and pro-inflammatory/anti-inflammatory ratio were significantly higher in adolescent AN girls compared to controls (*p* < 0.001, *p* = 0.042, *p* = 0.031, *p* = 0.021, *p* = 0.016, *p* = 0.001; respectively). The remaining parameters showed no significant between-group differences. All results are summarized in Table 1.

**Table 1:** Comparison of Circulating cytokines, cytokine profiles, and cytokine ratios between AN and control adolescent girls

Parameter	Controls (*N* = 35)	AN (*N* = 25)	Statistic	df	Effect size (Cohen´s d)	*p*-value
***Selected cytokines***						
IL-1α (pg/mL)	0.25 (0.19, 0.32)	0.17 (0.11, 0.20)	249.5	58	-0.51	**0.005**
IL-1β (pg/mL)	1.36 (0.91, 1.62)	1.95 (1.32, 2.76)	234.5	58	0.69	**0.002**
IL-2 (pg/mL)	1.76 (1.14, 3.05)	1.58 (1.00, 2.25)	360.5	58	-0.12	0.251
IL-4 (pg/mL)	2.18 (1.83, 2.72)	1.74 (1.29, 2.40)	327.0	58	-0.12	0.099
IL-6 (pg/mL)	0.81 (0.68, 1.18)	1.31 (0.84, 2.62)	263.0	58	0.63	**0.009**
IL-8 (pg/mL)	3.51 (2.40, 6.11)	2.89 (2.75, 5.07)	422.5	58	-0.08	0.828
IL-10 (pg/mL)	0.81 (0.66, 1.03)	0.66 (0.49, 1.00)	362.5	58	0.25	0.171
IFN-γ (pg/mL)	0.37 (0.27, 0.50)	0.46 (0.26, 0.96)	363.0	58	0.49	0.267
TNF-α (pg/mL)	2.36 (2.05, 2.75)	3.04 (2.03, 4.71)	293.5	58	0.74	**0.031**
***Cytokine profiles***						
Th1 profile	−0.22 ± 1.15	0.16 ± 1.76	1.01	58	0.26	0.319
M1 profile	−1.28 ± 1.48	0.76 ± 2.73	3.66	58	0.96	**< 0.001**
pro-inflammatory profile	−1.37 ± 4.03	0.98 ± 4.52	2.08	58	0.54	**0.042**
anti-inflammatory profile	0.36 (− 0.38, 0.89)	−0.81 (− 1.18, 0.86)	332.0	58	-0.18	0.116
***Cytokine ratios***						
Th1/Th2 ratio	−0.43 ± 1.43	0.31 ± 1.15	1.90	58	0.50	**0.031**
Th1/Treg ratio	−0.03 (− 0.32, 0.15)	0.38 (− 0.15, 0.87)	337.0	58	0.26	**0.021**
Th1/Th2 + Treg ratio	−0.32 (− 1.01, 0.14)	0.56 (− 0.44, 1.29)	293.0	58	0.43	**0.016**
pro-inflammatory/anti-inflammatory ratio	−0.99 (− 2.39, − 0.003)	0.88 (− 0.58, 2.42)	262.0	58	0.75	**0.001**

AN - anorexia nervosa, IL- interleukin, IFN-γ - interferon-gamma, TNF-α - tumor necrosis factor-alpha, Th1 - T helper 1 lymphocytes, M1 - macrophages M1, Th2 - T helper 2 lymphocytes, Treg - T regulatory lymphocytes. Cytokine profiles and ratios were calculated from z standardized data of individual logarithmically-transformed cytokine levels. Values are expressed as mean ± SD or median (interquartile ranges). A value of *p* ≤ 0.05 (in bold) was considered statistically significant

### Correlation analysis of the association between depressive symptoms and cytokines, cytokine profiles, and cytokine ratios

Firstly, CDI total score was significantly higher in adolescent AN girls compared to control girls (21.7 ± 10.5 vs. 8.8 ± 5.8; *p* < 0.001). The correlation analysis revealed no significant associations between CDI and individual cytokines, cytokine profiles, and cytokine ratios in adolescent AN girls .

### Correlation analysis of the association between BMI and cytokines, cytokine profiles, and cytokine ratios

No significant correlation was found between BMI score and all evaluated variables in adolescent AN girls.

### Regression analysis – impact of BMI on cytokines´ levels in anorexia nervosa

The regression analysis revealed a significant association between BMI and IL-4 and IL-1β in adolescent AN girls (Table 2).

**Table 2:** Estimated relationships between BMI and cytokines in anorexia nervosa

	95% Confidence Interval						*P*
	Estimate	SE	Lower	Upper	Odds ratio	z	
(Intercept)	16.2049	0.370	15.479	16.93045	1.09e + 7	43.772	< 0.001
IL-1α (pg/ml)	0.6175	4.125	-7.468	8.70279	1.854	0.150	0.882
IL-1β (pg/ml)	-1.1645	0.555	-2.252	-0.07742	0.312	-2.100	**0.046**
IL2 (pg/ml)	0.6237	0.429	-0.217	1.46466	1.866	1.454	0.158
IL4 (pg/ml)	1.2122	0.425	0.379	2.04576	3.361	2.850	**0.009**
IL6 (pg/ml)	-0.2584	0.203	-0.656	0.13907	0.772	-1.274	0.214
IL8 (pg/ml)	-0.2465	0.131	-0.503	0.00985	0.782	-1.885	0.071
IL10 (pg/ml)	-0.0681	0.125	-0.314	0.17736	0.934	-0.544	0.591
IFN-γ (pg/ml)	0.0590	0.521	-0.962	1.08037	1.061	0.113	0.911
TNF-α (pg/ml)	0.4757	0.307	-0.126	1.07744	1.609	1.549	0.134

IL – interleukin, IFN- interferon, TNF- tumor necrosis factor, SE – standard error. A value of *p* ≤ 0.05 (in bold) was considered statistically significant

### Regression analysis – impact of CDI on cytokines´ levels in anorexia nervosa

The regression analysis revealed a significant association between CDI and IL-8 in adolescent AN girls (Table 3).

**Table 3:** Estimated relationships between CDI and cytokines in anorexia nervosa

	95% Confidence Interval						*P*
	Estimate	SE	Lower	Upper	exp(B)	z	
(Intercept)	21.714	1.678	18.43	25.003	2.69e + 9	12.941	< 0.001
IL-1α (pg/ml)	-22.179	18.698	-58.83	14.468	2.33e-10	-1.186	0.247
IL-1β (pg/ml)	0.829	2.514	-4.10	5.756	2.290	0.330	0.744
IL2 (pg/ml)	0.276	1.945	-3.54	4.088	1.318	0.142	0.888
IL4 (pg/ml)	1.147	1.928	-2.63	4.925	3.148	0.595	0.557
IL6 (pg/ml)	-0.976	0.919	-2.78	0.825	0.377	-1.062	0.298
IL8 (pg/ml)	-1.597	0.593	-2.76	-0.435	0.203	-2.694	**0.012**
IL10 (pg/ml)	-0.842	0.568	-1.95	0.271	0.431	-1.483	0.151
IFN-γ (pg/ml)	1.507	2.362	-3.12	6.137	4.515	0.638	0.529
TNF-α (pg/ml)	1.688	1.392	-1.04	4.415	5.407	1.213	0.237

IL – interleukin, IFN- interferon, TNF- tumor necrosis factor, SE – standard error. A value of *p* ≤ 0.05 (in bold) was considered statistically significant

### ROC curve analysis

**Fig. 2:**
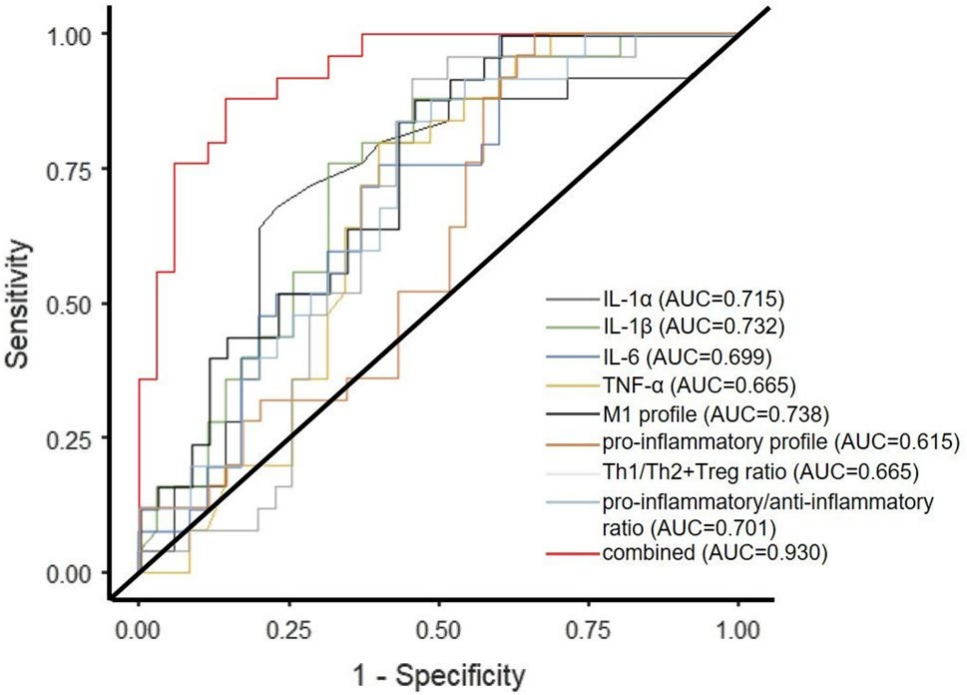
ROC curve analysis for anorexia nervosa diagnosis. AUC – area under the curve, IL – interleukin, M1 – macrophages M1, Th – T helper lymphocytes, Treg – T regulatory lymphocytes, TNF – tumor necrosis factor

The ROC analysis revealed different predictive abilities for significantly changed parameters between AN and control adolescent girls (i.e., AUC for IL-1α = 0.715, AUC for IL-1β = 0.732, AUC for IL-6 = 0.699, AUC for TNF-α = 0.665, AUC for the M1 profile = 0.738, AUC for the pro-inflammatory profile = 0.615, AUC for the Th1/Th2 + Treg ratio = 0.665, and AUC for the pro-inflammatory/anti-inflammatory ratio = 0.701). Moreover, the predictive ability of adolescent AN has been much improved through the combination of all parameters, which significantly differed between AN and control girls (AUC = 0.930). The ROC curve of the predictive value of IL-1α, IL-1β, IL-6, TNF-α, M1 profile, pro-inflammatory profile, Th1/Th2 + Treg ratio, and pro-inflammatory/anti-inflammatory ratio for MDD is presented in Fig. 2. Further, the AUC, sensitivity, and specificity comparisons are shown in Table 4.

**Table 4:** AUC, sensitivity, and specificity characteristics of selected parameters

	AUC	95%CI	Sensitivity	Specificity
IL-1α	0.715	0.586, 0.844	0.680	0.771
IL-1β	0.732	0.606, 0.858	0.680	0.686
IL-6	0.699	0.567, 0.831	0.640	0.629
TNF-α	0.665	0.528, 0.802	0.600	0.657
M1 profile	0.738	0.614, 0.862	0.640	0.657
pro-inflammatory profile	0.615	0.472, 0.758	0.520	0.514
Th1/Th2 + Treg ratio	0.665	0.528, 0.802	0.600	0.629
pro-inflammatory/anti-inflammatory ratio	0.701	0.570, 0.832	0.600	0.629
combination	0.930	0.865, 0.995	0.840	0.857

AUC – area under the ROC curve, CI – confidence interval, IL – interleukin, TNF – tumor necrosis factor, M1 – macrophages M1, Th – T helper lymphocyte, Treg – T regulatory lymphocyte

An overall DeLong test for the difference between dependent AUCs was statistically significant (*p* = 0.021), suggesting that there are meaningful differences in predictive ability among at least some of the models tested. Significant differences were identified between IL-1β and pro-inflammatory profile (Difference = 0.117; 95% CI: 0.007 to 0.227; *p* = 0.037). Furthermore, M1 profile was significantly better than pro-inflammatory profile (Difference = 0.123; 95% CI: 0.053 to 0.194; *p* = 0.001), and pro-inflammatory/anti-inflammatory ratio was significantly better than pro-inflammatory profile (Difference = -0.086; 95% CI: -0.155 to -0.016; *p* = 0.016). No other pairwise comparisons showed a statistically significant difference, including the comparison between the top two models (IL-1β vs. M1 profile, *p* = 0.896).

### Cross-validation analysis of the combined model AUC

A KNN classification method was employed to differentiate between AN patients and control probands with optimal configuration using k = 2 nearest neighbors, Euclidean distance, and rectangular weighting. The dataset was divided into 32 training, 16 validation, and 12 test samples. The model achieved a validation accuracy of 0.750 and a notably higher test accuracy of 0.917, indicating strong generalization performance on unseen data. Next, the class proportions were balanced across both datasets, with the AN class representing 58.3% of the training/validation set and 66.7% of the test set. The control class accounted for 41.7% and 33.3% in the respective splits. This relative balance minimizes concerns over potential class imbalance biasing the model. The confusion matrix indicated that the model correctly classified 58% of the AN cases and 33% of the control cases in the test set. Importantly, there were no false positives for the control class, meaning no control subject was misclassified as AN. Only 8% of AN cases were incorrectly labelled as control, suggesting that the model is more conservative when classifying the control group. Across both classes, the model achieved a high overall accuracy of 0.917. The precision was 1.000 for the AN class and 0.800 for the control class, indicating that the model produced very few false positives, particularly for the AN group. Recall was 0.875 for AN and 1.000 for control, suggesting that nearly all control cases were successfully identified, with only minimal misclassification of AN cases. The harmonic mean of precision and recall score (F1 score), a balance between precision and recall, was 0.933 for AN and 0.889 for control, with a macro-average of 0.919, confirming strong class-level performance. The Matthews Correlation Coefficient (MCC) was 0.837, reflecting a high level of agreement between predicted and true class labels. Notably, the AUC was 1.000 for both classes, demonstrating perfect separability in the decision space generated by the classifier. The model exhibited a false positive rate of 0.000 for AN and 0.125 for control, alongside an overall false omission rate of 0.100, further emphasizing the model’s reliability in minimizing incorrect exclusions of positive cases. The true negative rate remained high across both classes (AN: 1.000, control: 0.875), reinforcing the model’s robustness in correctly identifying non-target cases. To sum up, the KNN model demonstrated excellent discriminative ability with high precision, recall, and AUC scores. The absence of false positives for the AN class and strong F1 and MCC values indicate that the model reliably distinguishes between AN and control participants, making it a suitable candidate for further clinical or diagnostic applications.

## Discussion

The main significant findings of this case-control study can be summarized as follows: (1) increased levels of IL-1β, IL-6, TNF-α in association with increased M1 profile, pro-inflammatory profile, Th1/Th2 + Treg ratio, and pro-inflammatory/anti-inflammatory ratio in adolescent AN girls compared to controls; (2) decreased level of IL-1α in adolescent AN girls compared to controls; (3) an effect of BMI on the plasma levels of IL-4 and IL-1β in adolescent AN girls; (4) the effect of depressive symptomatology on the plasma level of IL-8 in adolescent AN girls; (5) a certain predictive value of significantly changed cytokines, cytokine profiles, and cytokine ratios in adolescent AN, which markedly improved by the combination of these parameters.

### Peripheral inflammation in anorexia nervosa

Systemic inflammation is considered to play an important role in the psychiatric disorders including AN which has been associated with alterations in the levels of various inflammatory markers [31], predominantly cytokines. Cytokines are cell signalling molecules produced by a wide range of cells (e.g. macrophages, lymphocytes, etc.) regulating immune responses [32]. These important signalling molecules are supposed to be implicated in AN pathogenesis due to their role in body weight [33–35], regulation of appetite and feeding behaviour [36, 37], and psychological well-being [4]. In this context, our study revealed increased levels of pro-inflammatory cytokines IL-1β, IL-6, and TNF-α in association with increased M1 profile, pro-inflammatory profile, Th1/Th2 ratio, Th1/Treg ratio, Th1/Th2 + Treg ratio, and pro-inflammatory/anti-inflammatory ratio indicating the activation of the inflammatory response system in AN adolescent girls. Several mechanisms are supposed. Cytokines can induce the release of hormones (e.g. leptin, cholecystokinin, and insulin), metabolic changes, and brain mechanisms modulating feeding regulation [38, 39]. Regarding individual pro-inflammatory cytokines significantly altered in our AN adolescent girls, IL-1β represents one of the major regulators of the inflammatory response produced predominantly by monocytes and macrophages and involved in IL-2 induction, IL-2 receptor production, IFN-γ synthesis stimulation by T lymphocytes, IL-6 synthesis stimulation by macrophages, stimulation of B cells proliferation and differentiation, and others [40]. Moreover, IL-1β plays an important role in modulation of hormonal activity influencing feeding behaviour [41, 42]. IL-6 represents a pleiotropic cytokine produced mainly by monocytes and macrophages which regulates defence mechanisms in the organism by primary action on B cells differentiating them into cells producing immunoglobulins of different classes. IL-6 is a crucial stimulator of the production of acute phase proteins, secretion of vascular endothelial growth factor, and lipolysis and glucose production in the liver [43]. TNF-α represents one of the main acute phase reaction cytokines produced primarily by monocytes and macrophages. TNF-α enhances the proliferation of B and T cells and differentiation of B cells, increases the cytotoxicity of monocytes and macrophages, and activates neutrophils by acceleration of their release from the bone marrow [8]. These findings are in line with previous meta-analysis reporting increased levels of the same pro-inflammatory cytokines (i.e. IL-1β, IL-6, and TNF-α) in adolescent and adult AN females [5]. However, more recent meta-analyses reported different conclusions. While Dalton et al. reported increased levels of IL-6 and TNF-α in adolescent and adult AN females [4], Maunder et al. reported increased level of only IL-6 in AN individuals [3]. Therefore, further research is needed to identify the precise AN-linked cytokines´ alterations. On the other hand, our study showed that the plasma level of another, but so far rarely studied pro-inflammatory cytokine, IL-1α, was decreased in AN adolescent girls, while previous studies reported its unchanged [7, 11] or increased levels [12]. These inconsistent findings warrant additional studies to elucidate the explicit role of individual cytokines in AN pathogenesis.

Additionally, cytokines´ plasma levels can be influenced by several factors including anthropometric variables such as BMI [17, 35]. However, the majority of the previous case-control studies regarding AN-inflammation relationship have not controlled the BMI effect in their analyses. Thus, it remains questionable if identified cytokines´ alterations are attributable to AN per se rather than simply BMI. From this perspective, in our study BMI significantly predicted plasma levels of two cytokines, pro-inflammatory IL-1β and anti-inflammatory IL-4. Their individual correlations with BMI suggest that adipose tissue or nutritional depletion can modulate these distinct immune axes in AN. On the other hand, our study revealed no significant correlations between BMI and the composite profiles/ratios. From this perspective, the construction of composite cytokine profiles and ratios often involves combining cytokines with diverse biological roles, variabilities, and regulatory mechanisms [44, 45]. Consequently, the integrative measure may fail to capture the nuanced effects of BMI on individual cytokine pathways. Moreover, BMI is the most commonly used marker of body mass, however, it may not fully capture the complexity of body composition and metabolic health [46]. In this context, composite cytokine profiles/ratios could be considered as more sensitive to these complexities than BMI alone, warranting further research in this area.

### Neuroimmune interactions and consequent neuroinflammatory modulation in anorexia nervosa

The bidirectional communication between the immune system and CNS and the reliance of each of these systems on intercellular communication molecules indicates that any alteration in one of the systems impacts the functioning of the another system [47]. Peripheral cytokines including IL-1β, IL-6, and TNF-α can directly affect the CNS through humoral pathway when cytokines access the CNS *via* leaky regions in blood brain barrier, neural pathway by changes in signalling from afferent nerves, and cellular pathway *via* facilitation and infiltration of activated peripheral cells (for a review see [48]). After reaching the CNS, cytokines can affect the mental state *via* several pathomechanisms including the impact of neuroinflammation on neurotransmitters´ signalling, neuroendocrine functions, and release of hormones modulating appetite and feeding [48–52]. Besides, an increased pro-inflammatory/anti-inflammatory ratio is suggested to be associated with a greater magnitude of neuropsychiatric manifestations because of deleterious cycles of cellular activation and cellular toxicity due to cytokine imbalance within CNS [38].

First of all, enhanced pro-inflammatory activity within CNS can affect the structure and function of hypothalamus as well as hypothalamic connections resulting in disturbed feeding behaviour and decreased body weight [6, 50]. Cytokines directly act on the feeding-associated sites of the hypothalamus such as neurons in the lateral hypothalamic area, the ventromedial nucleus, the paraventricular nucleus, and the arcuate nucleus [38]. Neurons in these areas of the hypothalamus express factors implicated in feeding and appetite regulation such as melanin-concentrating hormone, neuropeptide Y, proopiomelanocortin, orexin, and ghrelin [53]. Anorexic effects of pro-inflammatory cytokines including IL-1β, IL-6, and TNF-α can be mediated through the modulation of these appetite- and feeding-regulating hormones [4, 38, 54–56]. In addition, in the periphery cytokines can influence two critical factors involved in feeding regulation, leptin and ghrelin functioning *via* their receptors mainly through several hypothalamic nuclei [53].

Regarding neurotransmitters´ signalling, pro-inflammatory cytokines can lead to dysregulation of the synthesis, release, and re-uptake of serotonin, dopamine, and other neurotransmitters working within corticolimbic circuits associated with the core pathophysiology of AN [57, 58]. More specifically, neuroinflammation leads to the decreased dopamine signalling which can contribute to the rigid patterns of behaviour linked to AN [59] and to the tryptophan depletion impairing the production and release of serotonin [60]. Impaired serotoninergic metabolism within hypothalamus can lead to decreased food intake [61]. Moreover, pro-inflammatory cytokines within CNS can disrupt tetrahydrobiopterin (essential cofactor for enzymes involved in the synthesis of dopamine and serotonin) [62].

Regarding neuroendocrine functioning, pro-inflammatory cytokines can increase the activation of the hypothalamic-pituitary-adrenal (HPA) axis, likely through the inhibition of the glucocorticoid receptors [63]. This is of relevance, since HPA axis dysregulation has been observed in AN individuals, already at adolescent age [64].

Lastly, neuroinflammation is suggested to contribute to other AN-related symptoms, including fatigue, cognitive impairments, and sleep disturbances [31, 48].

### Impact of depressive symptomatology on inflammatory response in anorexia nervosa

It is important to note that immune dysregulation has been established in several psychiatric disorders, including depression [23, 65, 66] frequently co-occurring with AN [22, 31]. However, the relationship between AN, depressive symptomatology and inflammatory pathways is still not understood. In this context, our study found out an effect of depressive symptomatology on the level of IL-8 in AN adolescent girls. It means that the depressive symptomatology was negatively correlated with the level of pro-inflammatory marker which did not differ between AN and control adolescent girls. Similarly, the general psychopathology (depressive, anxiety, and stress symptoms) predicted levels of several inflammatory mediators including IL-8 in adult AN females [7]. Moreover, it is important to note that our previous study revealed a tendency to a significantly decreased levels of IL-8 in adolescent patients suffering from major depressive disorder compared to controls [23]. Although these findings can indicate a potential influence of depressive symptomatology on the AN-immune alterations´ relationship, further research is needed to fully understand the nature of this association.

### Strengths and limitations

One of the strengths of this study is the patient´s recruitment at the early stages of the disease in association with the absence of pharmacological treatment. In addition, all blood samples were drawn in the morning between 7 and 8 am to minimize the influence of a circadian rhythm on the cytokine production and release. On the other hand, several limitations of the study should be noted. The main limitation of the study is relatively small sample size which can limit the power of the study. Moreover, the discrepancy in the number of individuals in the studied groups (35 AN patients vs. 25 control probands) could introduce bias limiting the generalizability of the findings, and therefore the results should be interpreted with caution. Future research with larger and more balanced samples is warranted to confirm our findings. In addition, eating disorders including AN affect both, males and females. Since we were unable to recruit enough male patients, we have decided to evaluate only female patients which can affect the generalizability of the findings to AN males. Findings regarding AN adolescent males are almost lacking, therefore the sex-specific immune pathways in adolescent AN can be inferred from general sex-dependent differences in sex hormones production and immune regulation [67–69]. The sex hormones, including estrogen, progesterone, and testosterone, have been reported to influence immune functioning. Specifically, estrogen has been considered as pro-inflammatory or anti-inflammatory, depending on its levels and the immune context, while progesterone and testosterone have been described to be anti-inflammatory [67, 70]. From this perspective, during adolescence, particularly puberty, rising levels of sex hormones have been linked to changes in immune system activity. Emerging evidence suggests that these hormonal shifts may contribute to more pronounced changes in pro-inflammatory markers in females than in males, likely due to the immunomodulatory effects of estrogen [71]. This suggestion has been partially confirmed by a recent study revealing a significantly higher level of IL-6 in healthy adolescent females compared to males [72]. Moreover, the more advanced pubertal status was associated with higher CRP levels only among females. On the other hand, the more advanced pubertal status was associated with lower levels of TNF-α and IL-8 in both adolescent females and males [72]. With respect to AN, the so far single study comparing adolescent males and females revealed significantly higher IL-6 in females compared to males suffering from eating disorder generally, not specifically AN [73]. Besides hormonal between-sex differences, the body composition (fat vs. lean mass) divergence [74] can contribute to sex-linked distinct cytokine trajectories in AN adolescents. Thus, future research accounting for pubertal status, sex hormones, body composition, illness duration, and nutritional status is crucial for better understanding of the exact sex-related differences in inflammatory functioning in adolescent AN. Further, although this study compared AN girls and control girls to identify potential AN-related biomarkers, future studies should explore whether these potential biomarkers could distinguish AN adolescents from adolescent patients suffering from other mental disorders. Lastly, the cross-sectional design of the study limits the establishment of causal relationships between inflammation and AN.

## Conclusion

Anorexia nervosa in adolescent females in early stages of the disease and with no previous pharmacological treatment is characterized by enhanced IL-1β, IL-6, and TNF-α signalling indicating the activation of pro-inflammatory response system. These subtle dysregulations in inflammatory pathways can contribute to a clearer understanding of the complex immunopathogenesis of anorexia nervosa at adolescent age. However, further research is needed to identify precise anorexia nervosa-specific immune pathways, also with respect to sex and potential confounding factors.

## Data Availability

Data are available upon reasonable request from the corresponding author.

## Abbreviations

AN: Anorexia nervosa
AUC: Area under the curve
BMI: Body mass index
CDI: Children´s Depression Inventory
CNS: Central nervous system
HPA: Hypothalamic-pituitary-adrenal
IFN: Interferon
IL: Interleukin
M1: Macrophages M1
MDD: Major depressive disorder
ROC: Receiver Operating Characteristic
Th: T helper lymphocytes
TNF: Tumor necrosis factor
Treg: T regulatory lymphocytes
